# Migration of bisphenol A from commercially available pacifiers: HPLC-FLD analysis and exposure assessment in infants and toddlers

**DOI:** 10.1007/s11356-026-37444-1

**Published:** 2026-01-24

**Authors:** Lena Herwanger, Katharina Sternecker, Jan Kühnisch, Franz-Xaver Reichl, Christof Högg

**Affiliations:** 1 Department of Conservative Dentistry, Periodontology and Digital Dentistry, LMU University Hospital, LMU Munich, Goethestrasse 70, 80336 Munich, Germany; 2 Department of Anatomy II - Neuroanatomy, Faculty of Medicine, Ludwig-Maximilians-University of Munich, Munich, Germany

**Keywords:** Bisphenol A, Pacifiers, Chromatography, High pressure liquid, Chemical migration, Risk assessment

## Abstract

Bisphenol A (BPA) is an endocrine-disrupting compound widely used in plastics and resins and associated with metabolic, reproductive, and neurodevelopmental disorders. Infants and toddlers are particularly vulnerable because detoxification capacity is immature and exposure occurs during sensitive developmental stages. While BPA is banned in infant feeding bottles within the European Union, its use in pacifiers remains unregulated despite frequent “BPA-free” labeling. This study quantified BPA migration from seven commercially available pacifiers and assessed potential exposure relative to the newly revised European Food Safety Authority (EFSA) tolerable daily intake (TDI; 0.2 ng kg⁻^1^ bw day⁻^1^) in a worst-case exposure scenario. Pacifiers were dissected into shield and teat components, cut into fragments, and analyzed separately using validated high-performance liquid chromatography with fluorescence detection (HPLC-FLD). Measured BPA concentrations in the eluates (c(BPA,HPLC)) ranged from below the limit of quantification (LOQ) up to 288 µg/L. Based on these measured values, the extrapolated total BPA release per pacifier was 33 to 26,536 ng, with the highest migration observed in a “BPA-free” labeled product. Even the lowest total migration exceeded the 2023 EFSA TDI, whereas exposures would have been negligible under the former 2015 t-TDI (4 µg kg⁻^1^ bw day⁻^1^). These findings demonstrate that pacifiers can constitute a relevant early-life source of BPA exposure and contribute to already critical background levels. The results underline the unreliability of voluntary “BPA-free” claims and emphasize the need for harmonized EU regulation analogous to existing restrictions for feeding bottles and toys.

## Introduction

Bisphenol A (BPA) is one of the most extensively studied endocrine-disrupting chemicals and has been the focus of toxicological and regulatory attention for more than a decade. In 2016, BPA was classified by the European Chemicals Agency (ECHA) as toxic to reproduction and subsequently identified as a substance of very high concern under European chemicals legislation due to its endocrine-disrupting properties for humans and the environment (ECHA and European Chemicals Agency 2017). Endocrine disruptors are substances that interfere with hormonal signaling and may influence growth and developmental processes in organisms (WHO and UNEP 2013). Numerous studies have linked BPA exposure to severe systemic effects, including reproductive toxicity and infertility (Nomiri et al. 2019), metabolic and cardiovascular disorders such as obesity and type 2 diabetes (Zulkifli et al. 2021), and neurodevelopmental impairments (Nomiri et al. 2019). Moreover, experimental and epidemiological evidence suggests that BPA may interfere with enamel mineralization, contributing to conditions such as molar–incisor hypomineralization (MIH) (Jedeon et al. 2013). MIH is a multifactorial developmental enamel defect influenced by both genetic and environmental factors (Vieira and Manton 2019). Its etiology remains unclear and difficult to study, as ameloblasts are lost after tooth eruption and cannot be examined directly (Alaluusua 2010; Bandeira-Lopes et al. 2021; Crombie et al. 2009; Silvaet al. 2016). Because enamel formation occurs during a critical window in early childhood, exposure to endocrine-disrupting chemicals such as BPA during this period may interfere with amelogenesis. Supporting this rationale, recent experimental work demonstrated that BPA can disrupt biomineralization processes in zebra mussels (*Dreissena polymorpha*) (Liu et al. 2022), providing mechanistic plausibility that BPA may affect mineralization across species. Together, these findings underline the need to investigate BPA release from pacifiers as a possible contributor to early-life exposure.

BPA continues to be widely used in industrial applications. It serves as a key monomer in the production of polycarbonates (PC) and epoxy resins, and as an antioxidant additive in plasticizers, PVC, and flame retardants (EFSA and European Food Safety Authority 2021; German Environment Agency 2010). Epoxy resins are particularly common in food packaging, beverage containers, storage boxes, tableware, and medical devices, most prominently as protective linings in food cans. Several studies have demonstrated that BPA can migrate from such materials into food and beverages under realistic use conditions (Cao et al. 2010; Health 2010; Hoekstra and Simoneau 2013; Ni et al. 2023). Even trace concentrations may be toxicologically relevant given the controversially discussed low-dose effects (Dokmeci et al. 2022).

Infants and toddlers represent a particularly vulnerable population due to their immature hepatic metabolism and ongoing developmental processes (Nachman et al. 2015). BPA detoxification via glucuronidation is inefficient in early infancy, resulting in higher internal exposure to bioactive (unconjugated) BPA compared to adults (Doerge et al. 2011; Jalal et al. 2018). Migration from PC-based infant feeding bottles led the European Commission to ban such products in 2011, extending the prohibition to all drinking vessels for children under three years in 2018 (European Commission 2011, 2018).

However, the use of BPA in pacifiers—products that can remain in an infant’s mouth for several hours daily—remains legal within the EU, with the exception of Austria, which enacted a national ban in 2011 (Austrian Federal Ministry of Health 2011). Most pacifiers are marketed as “BPA-free,” yet this label is voluntary and not subject to independent verification. The shield components, typically composed of hard plastics, represent a potential source of BPA release through oral contact with saliva. Studies have shown that even short-term exposures under mild conditions can result in detectable migration (Kemi 2012; Lassen et al. 2011).

In 2023, the European Food Safety Authority (EFSA) drastically lowered the tolerable daily intake (TDI) for BPA from 4 µg/kg body weight/day to 0.2 ng/kg bw/day based on immunotoxicological endpoints (PoFCM EFSA et al. 2023). This represents a 20,000-fold reduction and places particular emphasis on low-dose effects during sensitive life stages, including the period of enamel formation linked to MIH. Given the absence of EU-wide regulation for BPA in pacifiers and the newly defined exposure limits, a reassessment of oral BPA sources for infants and toddlers is warranted.

The aim of the present study was to evaluate whether BPA is released from modern pacifiers and to what extent their routine use might contribute to BPA exposure in infants and toddlers. Seven commercially available pacifiers from different manufacturers were analyzed. Shield and teat components were tested separately using validated high-performance liquid chromatography with fluorescence detection (HPLC-FLD). Worst-case daily BPA uptake was estimated and evaluated against current toxicological thresholds. This study provides important data for consumer safety and regulatory decision-making concerning BPA exposure in early childhood.

## Materials and methods

### Sample selection and preparation

A convenience sample (as identical lots are rarely accessible through retail distribution channels) of seven pacifiers from different manufacturers in Germany, China, and India was investigated, representing a range of product types and geographical origins (Table 1). Products were acquired via retail and online platforms. Shield and teat components were analyzed separately for each model. One pacifier (ID 3) was unique in that both the shield and teat were made entirely of latex, whereas in all other models these components consisted of different materials. Given the exploratory nature of the study and the high analytical accuracy and reproducibility of the applied HPLC-FLD method, three independent measurements were performed for each component (*n* = 3), which were considered sufficient to provide a robust and reliable estimate of BPA migration (Stangl et al. 2025; Yang et al. 2019). Investigated pacifiers, including manufacturers’ information, are listed in Table 1. Material specifications for shields were generally not disclosed by manufacturers; thus, material assignments were based on product type and analytical inference where indicated.

**Table 1:** Investigated pacifiers: *n/a*, not available (information not provided by manufacturer)

ID	Manufacturer	Source	LOT	Shield material	Teat material	Label “BPA-Free”
1	NUK, Zeven, DE	Retail	0000659422	Hard plastic, transparent (colorless)	Latex	Yes
2	NUK, Zeven, DE	Retail	0000660992	Hard plastic, opaque (white)	Silicone	Yes
3	Hevea, Dragør, DK	Retail	19172	Latex		Yes
4	MMBABY, Bangalore, IN	Online	n/a	Hard plastic, opaque (yellow)	Silicone	No
5	Unknown, CN	Online	n/a	Hard plastic, opaque (green)	Silicone	No
6	FUNNYBABY, Unknown, CN	Online	2506	Hard plastic, transparent (black)	Silicone	No
7	Novatex, Pattensen, DE	Retail	494812	Hard plastic, opaque (green)	Latex	Yes

For toxicological assessment, migration results from shield and teat were combined numerically to represent the total pacifier. Only parts with plausible oral contact were considered. On shields, the ring, handle, and decorative elements were removed prior to fragmentation. Consequently, the effective masses used for risk assessment may have been lower than the nominal part weights, reflecting exposure-relevant material.

Each pacifier component was cut into approximately 2 × 2 mm fragments using chrome-plated side cutters (shields) or stainless-steel scissors (teats), yielding about 0.75 g of material per fraction. The fragments were incubated in 10-mL glass vials containing 2 mL methanol (GC Ultra Grade, ROTISOLV® ≥ 99.9%, Roth, Germany) at 37 °C for 72 h in the dark. After incubation, 1 mL of the eluate was evaporated to dryness under a gentle nitrogen stream and reconstituted in 600 µL of methanol/water (55:45, v/v). Samples were filtered through 0.2-µm centrifugal filters (Nanosep®, Pall, USA) at 13,200 rpm for 60 s. The resulting filtrates were subjected to HPLC-FLD analysis (injection volume: 100 µL).

All laboratory procedures were conducted under controlled conditions to minimize the risk of external contamination. Cleaned glassware was used throughout sample preparation, and BPA-free materials were employed whenever possible. Plastic laboratory ware was avoided throughout the entire sample preparation and analysis process.

### HPLC-FLD analysis

Quantitative analysis of BPA was performed using a LaChrom Elite® HPLC system (VWR, Germany) equipped with a fluorescence detector. Chromatographic separation was achieved on a LiChrospher 100 RP-18 column (5 µm, 250 mm × 4.6 mm, Merck) with a matching guard column. The mobile phase consisted of methanol and deionized water. The gradient started at 55% methanol, linearly increased to 75% over 24 min, then to 90% within 1 min and held for 5 min. It was returned to 55% within 1 min and equilibrated for 9 min. The flow rate was 0.7 mL/min. The detector was set at 270 nm (excitation) and 333 nm (emission) in accordance with the German Federal Institute for Materials Research and Testing (Federal Institute for Materials Research and Testing 2008).

BPA was identified by comparing retention times to those of external standards (14.24 min). Calibration was performed using BPA standard solutions at 1, 5, 10, 50, and 100 µg/L in methanol/water (55:45, v/v), each measured in six replicates. A linear calibration curve was constructed. BPA concentrations in pacifier samples were calculated accordingly and expressed as means ± standard deviation (SD).

Procedural blanks prepared from purified (deionized) water and processed in parallel with the samples showed no detectable BPA above the method detection limit.

### Method validation

Method validation was performed in accordance with the International Council for Harmonisation (ICH) guideline Q2(R2) (ICH and International Council for Harmonisation of Technical Requirements for Pharmaceuticals for Human Use 2023). The recovery rate was evaluated by matrix-spiking experiments at three concentration levels (*n* = 6). Repeatability was assessed from six replicate measurements of selected samples. The limit of detection (LOD) and limit of quantification (LOQ) were calculated from the calibration curve using the formulas LOD = 3.3 × σ/sl and LOQ = 10 × σ/sl, where σ is the standard deviation of the response and sl the slope of the calibration curve.

### Confirmation by GC-MS

Selected samples were analyzed by gas chromatography-mass spectrometry (GC-MS) to confirm BPA identity. A Finnigan Trace GC Ultra system coupled to a DSQ MS (Thermo Electron) with a J&W VF-5 ms column (30 m, 0.25 mm ID, 0.25 µm) was used. The PTV inlet was ramped from 30 to 320 °C (14.5 °C/s) and held for 5 min. Helium served as the carrier gas at 1 mL/min. The oven was held at 50 °C for 2 min, increased to 280 °C at 25 °C/min, and held for 5 min. The MS was operated in EI mode (70 eV) at 240 °C, and BPA was confirmed using full scan mode (m/z 50–600) and SIM mode (m/z 213 and 228). Identification was based on retention time and mass spectral match with a BPA reference standard.

### Calculation of BPA mass fraction in pacifier samples

For comparability with previous studies, BPA concentrations were also expressed as the mass fraction (ng/g) of the mass of BPA (m(BPA)) normalized to 1 g of pacifier material (shield or teat fragment m(S_fr_ or T_fr_)). The following equation was applied:

$$m(BPA)/m(S_{fr}\;or\;T_{fr})=\frac{\mathrm{c}(\mathrm{BPA,\;S_{HPLC}\;or\;T_{HPLC}})\times \mathrm{V_{ex}}\times \mathrm{V_{au}}}{\mathrm{V_{i}}\times \mathrm{m}(\mathrm{S_{fr}\;or\; T_{fr}})\times \mathrm{V_{ab}}\times \mathrm{DF}}[\mathrm{ng/g}]$$

m(S_fr_ or T_fr_): weighed mass of shield or teat sample fragment [g]
c(BPA,S_HPLC_ or T_HPLC_): BPA concentration in reconstituted solution, determined by HPLC-FLD [µg/L]
V_ex_: 2000 µL (extraction volume)
V_au_: 600 µL (reconstitution volume after evaporation)
V_i_: 100 µL (HPLC injection volume)
V_ab_: 1000 µL (eluate volume taken before evaporation)
DF: 1 (dilution factor; dimensionless)

### Statistical evaluation

Data were analyzed using Excel (Microsoft Corporation, Redmond, WA, USA) and SPSS 27.0 (IBM Corporation, Redwood, USA). For each sample group (*n* = 3), data distribution was assessed using the Shapiro–Wilk test (*p* > 0.05 for all datasets). Given the limited sample size and exploratory nature of the study, statistical power was inherently restricted, and results were therefore evaluated descriptively. Considering the low intra-assay variability and the high analytical precision of the applied HPLC–FLD method, values are reported as mean ± standard deviation (SD) to reflect measurement accuracy and variability.

## Results

### HPLC-FLD analysis

Calibration of the BPA reference standard using HPLC-FLD yielded a highly linear calibration curve with a coefficient of determination of *R*^2^ = 0.9999996:

$$y = 51444534x - 6577.75$$

**Fig. 1:**
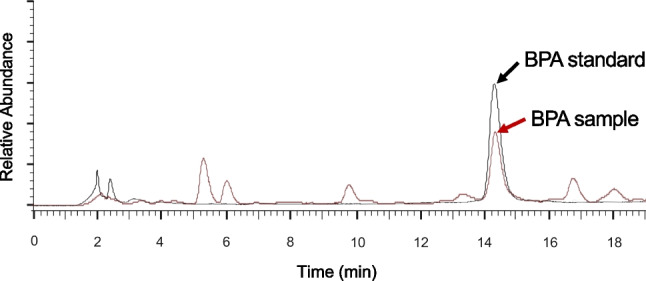
HPLC-FLD chromatograms demonstrating identical retention times for BPA reference standard (black) and sample extract (red)

**Fig. 2:**
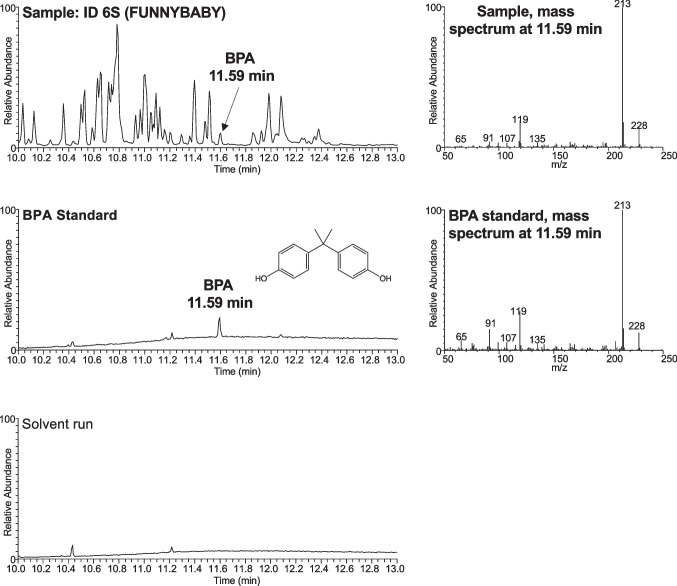
GC–MS chromatograms and mass spectra of sample ID 6S (shield) and the BPA reference standard, showing matching retention times and mass spectra, thereby confirming the compound’s identity

The method demonstrated a LOD of 0.22 µg/L and a LOQ of 0.66 µg/L. The mean recovery rate was 92.3%, and method repeatability, expressed as the relative standard deviation (RSD), was 2.41%. Chromatographic comparison of retention times between the BPA reference standard and the sample extract confirmed the presence of BPA (Fig. 1), and GC-MS analysis further verified the analyte identity by matching mass spectra (Fig. 2).

### BPA migration from shields and teats

BPA was detected in all investigated pacifier components, although some values in shield or teat fractions were below the LOQ (Tables 2 and 3). For shields, extrapolated total BPA masses ranged from 33 ng (ID 5S) to 26,536 ng (ID 6S). Migration normalized to 1 g of sample material varied between 11 ng/g and 4572 ng/g. Among the shield samples, only 2S yielded values below the LOQ. For teats, total BPA masses were generally lower, ranging from 33 ng (ID 2 T) to 467 ng (ID 7 T), with normalized values spanning from 27 ng/g to 289 ng/g. For teats, only 6 T values were below the LOQ.

**Table 2:** BPA migration results – pacifier shields: m(S_total_) = total shield mass; m(S_fr_) = weighed mass of shield sample fragment; c(BPA,S_HPLC_) = BPA concentration in reconstituted solution, LOD (0.22 µg/L) < x < LOQ (0.66 µg/L); m(BPA,S_inj_) = BPA mass per injection; c(BPA,S_total_) = extrapolated concentration for total shield; m(BPA)/m(S_fr_) = mass fraction of the mass of BPA relative to 1 g of shield; m(BPA,S_total_) = total BPA mass per shield

Pacifier ID Shield (S)		m(S_total_)	m(S_fr_)	c(BPA,S_HPLC_)	m(BPA,S_inj_)	c(BPA,S_total_)	m(BPA)/m(S_fr_)	m(BPA,S_total_)
		[g]	[g]	[µg/L]	[pg]	[µg/L]	[ng/g]	[ng]
1S NUK	mean	2.69	0.756	0.812	81.20	2.886	12.88	34.60
	SD		0.002	0.084	8.40	0.297	1.18	2.69
2S NUK	mean	3.17	0.757	x	-	-	-	-
	SD		0.002					
3S Hevea	mean	7.08	0.757	2.459	245.90	23.038	39.00	276.18
	SD		0.002	0.533	53.30	4.997	3.26	21.25
4S MMBABY	mean	4.87	0.757	1.345	134.50	8.666	21.31	103.83
	SD		0.001	0.175	17.50	1.125	3.23	14.62
5S unknown	mean	3.01	0.759	0.693	69.30	2.761	10.96	33.00
	SD		0.001	0.111	11.10	0.443	2.41	6.02
6S FUNNYBABY	mean	5.80	0.757	288.293	28,829.30	2213.3	4572.05	26,536.16
	SD		0.002	37.012	3701.20	284.151	586.22	3401.14
7S Novatex	mean	3.27	0.757	2.143	214.30	9.288	36.00	117.61
	SD		0.001	0.24	24.00	1.04	4.01	13.07

**Table 3:** BPA migration results – pacifier teats: m(T_total_) = total teat mass; m(T_fr_) = weighed mass of teat sample fragment; c(BPA,T_HPLC_) = BPA concentration in reconstituted solution, LOD (0.22 µg/L) < x < LOQ (0.66 µg/L); m(BPA,T_inj_) = BPA mass per injection; c(BPA,T_total_) = extrapolated concentration for total teat; m(BPA)/m(T_fr_) = mass fraction of the mass of BPA relative to 1 g of teat; m(BPA,T_total_) = total BPA mass per teat

Pacifier ID Teat (T)		m(T_total_)	m(T_fr_)	c(BPA,T_HPLC_)	m(BPA,T_inj_)	c(BPA,T_total_)	m(BPA)/m(T_fr_)	m(BPA,T_total_)
		[g]	[g]	[µg/L]	[pg]	[µg/L]	[ng/g]	[ng]
1T NUK	mean	1.627	0.758	2.274	227.41	4.896	35.99	58.55
	SD		0.001	0.414	41.43	0.414	7.44	11.39
2T NUK	mean	1.211	0.756	1.694	169.38	2.714	26.89	32.56
	SD		0.001	0.147	14.66	0.147	2.42	2.42
3T Hevea	mean	1.535	0.757	9.017	901.70	18.31	142.88	219.31
	SD		0.001	0.720	72.03	0.72	11.20	16.89
4T MMBABY	mean	3.494	0.756	6.306	630.60	10.042	100.14	349.89
	SD		0.001	0.625	62.51	0.626	10.17	34.94
5T unknown	mean	1.203	0.757	9.402	940.23	14.962	149.11	179.38
	SD		0.001	0.163	16.33	0.64	3.22	3.61
6T FUNNYBABY	mean	1.437	0.756	x	-	-	-	-
	SD		0.001					
7T Novatex	mean	1.617	0.756	18.208	1820.78	4.066	288.89	467.13
	SD		0.002	0.286	28.63	0.065	1.31	1.62

### Total BPA migration from complete pacifiers

**Fig. 3:**
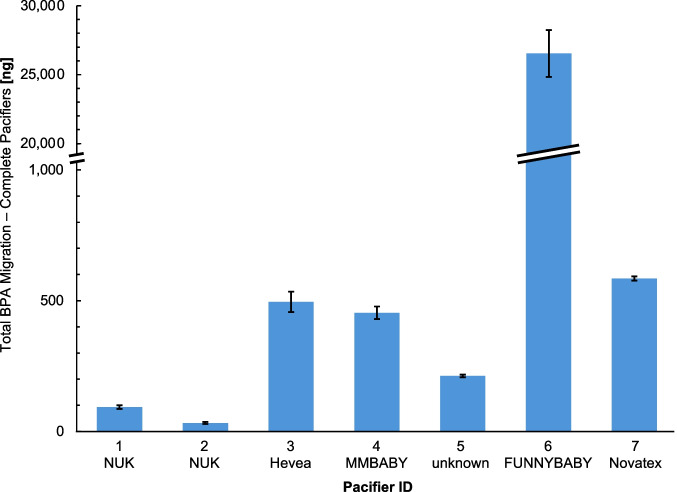
Total BPA migration from complete pacifiers (shield + teat). Bars represent mean values from shield and teat fractions; error bars indicate standard deviations

For comparability with previous studies and subsequent risk assessment, BPA migration data from shield and teat fractions were combined to calculate the total BPA migration per pacifier. Overall, BPA release ranged from 33 ng (ID 2) to 26,536 ng (ID 6). Except for this highest-migration pacifier (ID 6), all other models released < 1000 ng BPA in total (Fig. 3).

## Discussion

### Methodological considerations

The present study was designed to evaluate BPA migration from commercially available pacifiers under worst-case extraction conditions. BPA quantification was performed by HPLC-FLD, a method offering high sensitivity and selectivity for phenolic compounds such as BPA. Previous validation studies have demonstrated that HPLC-FLD achieves limits of detection and reproducibility within the same order of magnitude as LC-MS/MS (Yi et al. 2010), supporting its adequacy for low-level BPA analysis in biological and environmental matrices, as well as consumer products (Krivohlavek et al. 2023; Vilarinho et al. 2020). To strengthen analytical reliability, identification was independently confirmed by GC-MS without derivatization, matching both retention time and mass spectra with reference standards. The combined use of HPLC-FLD and GC-MS ensures a high level of analytical confidence.

In contrast to many earlier studies that tested pacifiers in their intact form (Kemi 2012; Lassen et al. 2011; Vicente-Martinez et al. 2020), the present study analyzed shield and teat separately, allowing the relative contribution of each component to BPA release to be identified. Methanol was selected as the extraction solvent to establish worst-case migration conditions. Its high affinity for hydrophobic phenolic compounds such as BPA, combined with its inability to dissolve the polymer backbone, makes it particularly suitable for exhaustive extraction (Gatidou et al. 2007; Polydorou et al. 2012; Wedekind et al. 2021). While water-based media better mimic oral conditions, they may promote hydrolytic degradation of BPA during prolonged incubation (Wang et al. 2021). In contrast, methanol enables maximum recovery of residual analytes without significant chemical breakdown (Wedekind et al. 2021) and has been widely applied in polymer and silicone extraction studies to enhance the release of organic additives (Padilla et al. 2005; Walther et al. 2019).

A 72-h extraction at 37 °C in the dark was applied to ensure exhaustive release of analytes while minimizing photodegradation. Although this far exceeds realistic use scenarios, it provides a conservative safety margin for toxicological risk assessment. Similar extraction protocols have been used in materials science and dental resin research to quantify maximum release under worst-case conditions, including comparative solvent studies with water, artificial saliva, and methanol (Rothmund et al. 2015). Taken together, this approach ensures that the measured BPA release reflects a worst-case scenario suitable for comparison with health-based guidance values such as the EFSA TDI. Percentile-based exposure estimates (e.g., 95th percentile) were beyond the scope of this materials-focused study, as they would require population-level data on pacifier use patterns and infant behavior. The present study focused exclusively on BPA, for which the analytical method was specifically calibrated and validated. Although certain BPA analogues such as bisphenol S (BPS) may in principle be detectable by HPLC-FLD, their quantification was beyond the validated analytical scope of this study and therefore not included.

### BPA migration in context of literature

The results of the present study revealed a broad range of BPA migration levels across the tested pacifier models. The pacifier ID 6 showed exceptionally high BPA migration from the shield component, with concentrations exceeding 288 µg/L (c(BPA,S_HPLC_)) under methanol extraction conditions. This finding is particularly relevant, as the product was marketed with a “BPA-free” label—an unregulated term in the EU that does not require third-party verification. Closer analysis revealed that this elevated release was confined to the shield, while the corresponding teat showed BPA concentrations below the LOQ. Among the remaining pacifiers, measured BPA concentrations in the eluates ranged from approximately 1.7–18 µg/L for teats and 0.7–2.5 µg/L for shields, indicating a general tendency toward higher migration from teat materials. Latex teats (IDs 1, 7) and silicone teats (IDs 2, 4, 5) exhibited overlapping concentration ranges, suggesting that the polymer type alone does not account for the observed variability. Pacifier ID 3—the only model made entirely of latex (shield + teat)—showed more than a threefold higher BPA concentration in the teat (9 µg/L) compared with the shield (2.5 µg/L). Such intra-material variation indicates that diffusion and migration behavior can vary substantially even within the same base material, likely influenced by differences in cross-linking density, curing additives, or surface treatment during molding—factors known to affect polymer permeability and additive migration (Fang and Vitrac 2017; George and Thomas 2001).

The high-migration shield of ID 6, despite its “BPA-free” label, further illustrates that labeling alone is not a reliable indicator. Previous studies have similarly reported detectable BPA migration from products advertised as “BPA-free” (Kemi 2012; Lassen et al. 2011). Richter and Simat (2010) found BPA concentrations between 0.1 and 2 µg/L in aqueous pacifier extracts, while Lassen et al. (2011) reported up to 8 µg/L in artificial saliva. The higher levels observed in the present study are consistent with the increased extraction efficiency of methanol, which represents a worst-case migration scenario.

Variability in BPA release between products likely reflects differences in polymer formulation, processing conditions, and contamination during manufacturing. PC, synthesized from BPA and phosgene, is a well-documented source of BPA release (Brede et al. 2003; Le et al. 2008; Vogel 2009), whereas polypropylene (PP) is generally considered BPA-free (Biedermann-Brem et al. 2008). Nevertheless, trace contamination can occur via BPA-based additives such as colorants, stabilizers, or coatings (Vandenberg et al. 2007), or through residual process contamination (Ighalo et al. 2024). In the present study, material information was not disclosed by manufacturers; visual and tactile classification suggested combinations of hard plastic (shields), silicone, and latex (teats). The markedly elevated BPA level in the shield of ID 6 is consistent with the possible use of PC, although this remains an analytical interpretation rather than a verified composition.

Measured BPA concentrations in teats ranged from below the LOQ to 18 µg/L, confirming that even polymers not based on BPA (silicone and latex) can release measurable levels. This observation agrees with literature reports attributing low-level BPA findings in non-PC materials to cross-contamination during processing, migration from colored coatings, or packaging contact (Biedermann-Brem et al. 2008; Geueke et al. 2014).

Beyond migration studies, biomonitoring investigations have demonstrated that such material emissions can translate into measurable internal exposure. Prado et al. (2025) detected BPA in saliva of children after contact with polymer-based oral devices, and Yi et al. () reported median BPA concentrations of 10.4 ng/mL in breast milk. These data provide biological plausibility that migration from child-contact materials contributes to systemic exposure in infants and toddlers.

Beyond migration studies, biomonitoring investigations demonstrate that infants are exposed to BPA via multiple pathways. Prado et al. (2025) detected BPA in the saliva of children after contact with polymer-based oral devices, indicating a direct contribution of child-contact materials to local exposure. In contrast, Yi et al. reported median BPA concentrations of 10.4 ng/mL in breast milk (Yi et al. 2010), reflecting maternal background exposure and transfer to the infant, rather than migration from child-contact materials. Taken together, these findings underscore that infants and toddlers may experience cumulative BPA exposure from both product-related migration and maternally mediated sources.

A temporal comparison with earlier market surveys also suggests progress toward lower BPA contamination. Global 2000 (Global 2000 2009) reported maximum BPA contents of 2284 mg/kg for shield components and 437 mg/kg for teats—values over 500-fold higher than those calculated in the present study (4.29 mg/kg for ID 6 shield, 0.15 mg/kg for ID 5 teat). This indicates a substantial shift toward low-BPA formulations, although isolated high-migration products such as ID 6 demonstrate that the issue has not been entirely eliminated.

Overall, the data reveal mostly low to moderate BPA release from current pacifiers, with individual outliers showing markedly higher migration. This heterogeneity underscores the need for continuous market surveillance and provides the rationale for the subsequent toxicological risk assessment.

### Risk assessment

Risk characterization was performed using both the 2015 EFSA t-TDI of 4 µg/kg bw/day (EFSA and European Food Safety Authority 2015) and the revised 2023 EFSA TDI of 0.2 ng/kg bw/day (PoFCM EFSA et al. 2023). The latter represents a 20,000-fold reduction and a shift from earlier assessments that primarily focused on endocrine-disrupting properties to immunotoxicological endpoints.

To capture the full range of possible exposure, two complementary calculations were conducted. As a representative case, the pacifier ID 6 with the highest overall BPA migration was selected. The first approach represents a worst-case scenario, assuming complete transfer and systemic absorption of all BPA released from both shield and teat within a single day, based on the 72-h methanol extraction extrapolated linearly to 24 h. Under these assumptions, an infant weighing 4.5 kg would be exposed to 1.97 µg/kg bw/day, and a toddler weighing 12 kg to 0.74 µg/kg bw/day. For contextualization, EFSA estimated a baseline daily BPA exposure of 0.36 µg/kg bw/day for infants and toddlers under worst-case conditions, without pacifiers (EFSA and European Food Safety Authority 2015). Adding the present migration results yields a combined potential intake of 2.33 µg/kg bw/day (infant) and 1.10 µg/kg bw/day (toddler). Compared with the 2015 t-TDI of 4 µg/kg bw/day (EFSA and European Food Safety Authority 2015), these intakes remain below the health-based limit (1.7-fold lower for infants, 3.6-fold lower for toddlers). Under the revised 2023 EFSA TDI of 0.2 ng/kg bw/day (PoFCM EFSA et al. 2023), they exceed the threshold by ~ 11,600-fold (infant) and ~ 3700-fold (toddler).

In a second step, to better approximate physiological conditions, the exposure model by EFSA was applied (EFSA and European Food Safety Authority 2015). This model accounts for daily saliva production (0.2 L/day infants, 0.3 L/day toddlers), pacifier use duration (4.8 h/day), and the proportion of material surface in oral contact (25% shield, 100% teat) (Bremmer and van Veen 2002; EFSA and European Food Safety Authority 2015; Lassen et al. 2011). Applying this approach to the pacifier with the highest migration (ID 6) resulted in estimated intakes of 0.38 µg/kg bw/day for a 4.5-kg infant and 0.18 µg/kg bw/day for a 12-kg toddler. Relative to the 2015 t-TDI (EFSA and European Food Safety Authority 2015), these values are still well below the threshold (tenfold lower for infants, 22-fold lower for toddlers). Under the 2023 TDI (PoFCM EFSA et al. 2023), however, they exceed the limit by ~ 1900-fold (infant) and ~ 900-fold (toddler).

For the lowest-migration pacifier ID 2, applying the same assumptions, intakes were 0.010 µg/kg bw/day (infant) and 0.0049 µg/kg bw/day (toddler). These values remain more than 400-fold (infant) and 800-fold (toddler) lower than the 2015 t-TDI (EFSA and European Food Safety Authority 2015), while exceeding the 2023 TDI (PoFCM EFSA et al. 2023) by ~ 50-fold and ~ 25-fold, respectively. Notably, EFSA’s baseline dietary exposure estimate of 0.36 µg/kg bw/day for infants and toddlers (EFSA and European Food Safety Authority 2015) already exceeds the 2023 TDI by more than 1000-fold, indicating that even without pacifier use, background exposure alone surpasses the updated health-based guidance value. Pacifiers—even at the lowest migration levels observed—therefore represent an additional and relevant contribution to an already critical exposure margin.

While the present data allow estimation of absolute BPA intake from pacifier use, a precise quantification of the relative percentage contribution of pacifiers to total BPA exposure is not feasible. Such an assessment would require detailed population-level data on pacifier use duration, sucking behavior, feeding practices (breastfed vs. non-breastfed), and concurrent exposure sources, which are currently not available. The objective of this study was therefore not to rank pacifiers among all exposure pathways, but to determine whether pacifiers can represent a non-negligible additional source of BPA exposure under conservative assumptions. In light of the markedly reduced EFSA TDI, even quantitatively small additional inputs may be relevant from a regulatory and precautionary risk assessment perspective, as they further reduce the margin between background exposure and health-based guidance values.

Nevertheless, physiological conditions (aqueous media, intermittent contact, shorter exposure duration) are expected to yield substantially lower migration values than methanol extraction. Accordingly, the calculated exposures should be regarded as conservative upper-bound estimates rather than realistic in vivo predictions. Importantly, while such exposure would have been negligible in relation to the 2015 t-TDI, it now constitutes a clear exceedance under the 2023 TDI. This regulatory shift underscores that even low-level BPA migration from pacifiers can no longer be dismissed as toxicologically irrelevant. From a risk perspective, the presence of high-migration products is of particular concern, as infants and toddlers are the most vulnerable and may already be exposed to BPA from multiple sources.

Beyond systemic considerations, potential dental implications also merit attention. Experimental data indicate that BPA can interfere with enamel biomineralization: perinatal BPA exposure in rats produced enamel defects via estrogen receptor–mediated pathways (Jedeon et al. 2013), and Liu et al. () demonstrated BPA-induced disruption of shell biomineralization in Dreissena polymorpha, a model used to screen for factors linked to MIH. While these findings are not directly translatable to humans, they support mechanistic plausibility that early-life BPA exposure could coincide with sensitive periods of amelogenesis.

Importantly, the finding that a “BPA-free” labeled pacifier ID 6 released substantial amounts of BPA highlights the unreliability of voluntary labeling without binding definitions or independent verification. This underscores the need for harmonized EU-level regulation of pacifiers, comparable to the restrictions already in place for feeding bottles and toys.

## Conclusion

This study demonstrates that pacifiers can constitute a relevant early-life source of BPA exposure. While most products showed low migration, individual models released markedly higher amounts, including one labeled as “BPA-free.” Importantly, even the lowest migration levels observed exceeded the latest EFSA TDI. These results underline the need for harmonized regulatory standards and independent verification of “BPA-free” claims to ensure adequate protection of infants and toddlers.

## Data Availability

The data supporting the findings of this study are included in this article. Additional raw data files are available from the corresponding author upon reasonable request.
